# Magnesium Deficiency Questionnaire: A New Non-Invasive Magnesium Deficiency Screening Tool Developed Using Real-World Data from Four Observational Studies

**DOI:** 10.3390/nu12072062

**Published:** 2020-07-11

**Authors:** Svetlana Orlova, Galina Dikke, Gisele Pickering, Sofya Konchits, Kirill Starostin, Alina Bevz

**Affiliations:** 1 Department of Dietetics and Clinical Nutritiology of Continuing Medical Education, Medical Institute, RUDN University, 119571 Moscow, Russia; rudn_nutr@mail.ru; 2 Department of Obstetrics and Gynecology with a Course of Reproductive Medicine, The Academy of Medical Education F.I. Inozemtsev, 190013 Saint-Petersburg, Russia; galadikke@yandex.ru; 3 Department of Clinical Pharmacology, University Hospital and Inserm 1107 Fundamental and Clinical Pharmacology of Pain, Medical Faculty, F-63000 Clermont-Ferrand, France; gisele.pickering@uca.fr; 4 Medical Affairs, Sanofi, 125009 Moscow, Russia; sofya.konchits@sanofi.com (S.K.); alina.bevz@sanofi.com (A.B.)

**Keywords:** hypomagnesemia, magnesium deficiency questionnaire, pregnancy, hormone-related conditions

## Abstract

Due to the high estimated prevalence of magnesium deficiency, there is a need for a rapid, non-invasive assessment tool that could be used by patients and clinicians to confirm suspected hypomagnesemia and substantiate laboratory testing. This study analyzed data from four large observational studies of hypomagnesemia in pregnant women and women with hormone-related conditions across Russia. Hypomagnesemia was assessed using a 62-item magnesium deficiency questionnaire (MDQ-62) and a serum test. The diagnostic utility (sensitivity/specificity) of MDQ-62 was analyzed using area under the receiver operating characteristic curve (AUROC). A logistic regression model was applied to develop a shorter, optimized version of MDQ-62. A total of 765 pregnant women and 8836 women with hormone-related conditions were included in the analysis. The diagnostic performance of MDQ-62 was “fair” (AUROC = 0.7−0.8) for women with hormone-related conditions and “poor” for pregnant women (AUROC = 0.6−0.7). The optimized MDQ-23 (23 questions) and MDQ-10 (10 questions) had similar AUROC values; for all versions of the questionnaire, there was a significant negative correlation between score and changes in total serum magnesium levels (*p* < 0.0001 for all comparisons; correlation coefficients ranged from −0.1667 to −0.2716). This analysis confirmed the value of MDQ in identifying women at risk of hypomagnesemia.

## 1. Introduction

Magnesium deficiency and low magnesium intake are associated with altered levels of other electrolytes, cardiovascular events, various metabolic and neuromuscular conditions, type II diabetes mellitus, and depression [1]. Studies of various populations showed that 15–42% of apparently healthy adults have subnormal serum magnesium levels; magnesium deficiency is more frequent in women than in men, and the proportions are much higher in post-menopausal women and in individuals with obesity or type 2 diabetes [1,2]. 

Despite its implications in clinical practice, diagnosing magnesium deficiency still presents a challenge. To date, there is no gold standard for how to determine magnesium levels. Most of the magnesium tests used in research, such as 24-hour urine magnesium load test, muscle and bone concentration measurements, nuclear magnetic resonance imaging, and isotope studies, are considered impractical in the clinic [3]. The measurement of total magnesium concentration in serum using spectrophotometry with titan yellow or xylidyl blue is the most practical and reliable approach; however, there is an argument that the results can only serve as an approximation of magnesium concentrations in tissues [1,2,3]. The optimal cut-off serum concentration that indicates magnesium deficiency is also a matter of ongoing debate. In Russia and other countries, the most commonly used lower reference limit is 0.66 mmol/L [4]. However, the largest historical study in the US identified 0.75 mmol/L as the reference limit, and recent publications suggest that the cut-off should be set at 0.8 mmol/L [2,3,5].

Only about 0.8% of total body magnesium is present in blood, with the rest stored in soft tissue (19%), muscle (27%), and bone (53%), meaning that magnesium deficiency can be present even when serum magnesium levels are in the normal range [1,3,6,7,8]. Conversely, a low serum magnesium level is a clear indication of overall magnesium deficiency. However, due to the lack of specific symptoms, magnesium deficiency is rarely suspected in the clinic, and furthermore, serum levels or other tests (e.g., magnesium level in red blood cells) may not be reimbursed [3]. For these reasons, in many countries including Russia, magnesium tests are rarely reimbursed by insurance companies, emphasizing the need for a simple, reliable, and affordable screening tool to help identify magnesium deficiency.

Magnesium deficiency can be suspected and diagnosed with the help of the ‘magnesium deficiency questionnaire’ (MDQ-62), consisting of 62 questions that can be grouped into 5 general categories: wellbeing, lifestyle, pregnancy, disease, and medication [9]. Although MDQ-62 may help identify non-specific symptoms frequently accompanying magnesium deficiency, the questionnaire is cumbersome and time-consuming, and several questions are very similar. It is currently unclear to what extent MDQ-62 scores correlate with total magnesium serum concentration and whether it can be a reliable surrogate for laboratory values.

Four large observational studies conducted between 2012 and 2016 across multiple regions and cities in the Russian Federation assessed the prevalence and clinical management of magnesium deficiency in pregnant women (MAGIC, MAGIC2) and in women with hormone-related conditions (MAGYN, MAGYN2) using MDQ-62 and laboratory tests [10,11,12,13]. Here, we report the results of a secondary analysis using pooled data from these studies, designed to describe the prevalence of magnesium deficiency in these populations and to identify associated risk factors and comorbidities. Another key objective of this analysis was to develop shorter, optimized versions of the questionnaire that would offer the same level of accuracy in identifying suspected hypomagnesemia.

## 2. Materials and Methods

This manuscript summarizes a part of the secondary analysis of pooled data collected in four observational studies of magnesium deficiency in pregnant women and women with hormone-related conditions: MAGIC (DIREGL06157), MAGIC2 (DIREGL06468), MAGYN (MAGNEL06863), and MAGYN2 (MAGNEL07741) [10,11,12,13].

MAGIC and MAGIC2 enrolled pregnant women (*N* = 1130 and *N* = 2117, respectively) during routine visits to maternity welfare centers. Women were included in the studies if they were >18 years of age, were pregnant, and had suspected magnesium deficiency (fatigue, muscle cramps, etc.). The study excluded women who reported other known or obvious reasons for magnesium deficiency beside pregnancy [11,13]. MAGYN and MAGYN2 studies enrolled women with hormone-related conditions (*N* = 9168 and *N* = 11,424, respectively) attending outpatient clinics. Women were included if they were 18–60 years of age and used hormonal contraception or hormone replacement therapy (HRT) or had one of the following conditions: premenstrual syndrome (PMS), climacteric syndrome without HRT, osteoporosis, or other hormonal conditions (including endometriosis, polycystic ovarian disease, uterine leiomyoma, algodysmenorrhea, endometrial hyperplastic processes). Women were excluded if they had severe conditions potentially hindering their participation in the study or were receiving magnesium supplementation at baseline [10,12].

**Figure 1:**
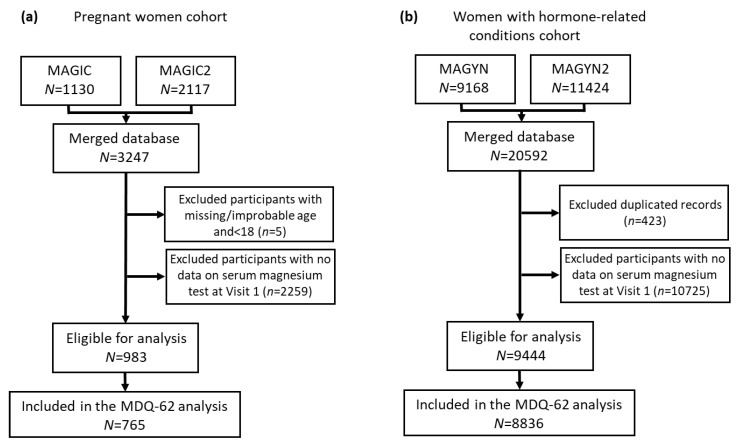
Study population: (**a**) Pregnant women cohort; (**b**) Women with hormone-related conditions cohort.

The present analysis included all patients who fulfilled the inclusion/exclusion criteria in the studies (Figure 1). Patients with missing data, contradictory/inconsistent data, or outlier data were excluded from the analysis (exclusion was performed separately for each variable of interest). Pooled databases were created for patient populations of “pregnant women” (MAGIC and MAGIC2) and ‘women with hormone-related conditions’ (MAGYN and MAGYN2).

### 2.1. Study Visits and Treatment

MAGIC, MAGIC2, MAGYN, and MAGYN2 were observational studies; during these studies, all treatment decisions were made by the treating physicians. 

Epidemiological data were collected at baseline (Visit 1) for all participants. Patients with low serum magnesium at this visit or with suspected deficiency based on MDQ-62 attended Visit 2 and underwent a second assessment of serum magnesium. Visit 2 was scheduled by the treating physicians according to their standard practice; in MAGYN and MAGYN2, it occurred approximately 4 weeks after Visit 1.

The analysis of the effectiveness of magnesium supplementation included participants who had mild hypomagnesemia at Visit 1 (serum levels above 0.5 mmol/L but below 0.66 mmol/L or 0.8 mmol/L, depending on the specific cut-off used) and who were prescribed a combination of magnesium and vitamin B6, Magne B6^®^ or Magne B6 Forte^®^ (Sanofi), for approximately 4 weeks. Patients who received other types of magnesium supplementation were excluded from the analysis.

### 2.2. Study Objectives and Endpoints

The objectives of this secondary analysis were (1) to assess the sensitivity and specificity of MDQ-62; (2) to develop a shortened version of MDQ using regression modelling; (3) to assess the sensitivity and specificity of this new shortened version of MDQ, and (4) to analyze the ability of MDQ and shortened MDQ to reflect dynamic changes in serum magnesium level.

The following research questions were assessed.

Question 1: What are the sensitivity and specificity of MDQ-62 when detecting hypomagnesemia in women with hormone-related conditions, using serum magnesium level cut-offs of 0.66 mmol/L or 0.8 mmol/L?

Question 2: What are the sensitivity and specificity of MDQ-62 when detecting hypomagnesemia in pregnant women, using serum magnesium level cut-offs of 0.66 mmol/L or 0.8 mmol/L?

Question 3: Can the number of questions in MDQ be reduced without a loss of diagnostic quality?

Question 4: Is it possible to detect changes in the serum magnesium level using MDQ-62 or shortened MDQ?

### 2.3. Questionnaires

The MDQ-62, consisting of 62 questions, was developed previously and adapted for use in the Russian Federation [9,11]. Each question contributed 2–5 points to the overall score. A score of 51 points or more was used as an indication that magnesium deficiency was ‘highly probable’, whereas a score of 30–50 points was interpreted as ‘likely’ magnesium deficiency [9,11].

To optimize the questionnaire, each of 62 questions was tested for contribution to the total MDQ score as well as for association with the magnesium level using the population of women with hormone-related conditions as the training sample (see Statistical analysis for further details). Questions independently associated with hypomagnesemia were selected and assembled in the modified MDQ. The modified shortened MDQ was tested on the pregnant women cohort (testing sample).

The diagnostic performance of MDQ-62 and modified MDQ was analyzed in women who had serum magnesium level data and questionnaire data at Visit 1.

### 2.4. Statistical Analysis

The sensitivity and specificity of MDQ-62 were analyzed using area under the receiver operating characteristic curve (AUROC), MDQ cut-off, positive and negative predictive values, and the likelihood ratio. Statistical significance of AUROC was analyzed using a Mann–Whitney test.

For the development of the modified MDQ, stepwise multiple regression was applied to obtain a shortened version of the questionnaire. All 62 questions of MDQ-62 were included and excluded into the testing empty model step by step, using a bidirectional stepwise selection approach and assessing explanatory capabilities each time after inclusion or exclusion of a variable with the following parameters or variable to select and to keep: stepwise slEntry = 0.00001, slStay = 0.1, accordingly (software SAS version 9.4, SAS Institute Inc., Cary, NC, USA). The model was developed for both cut-offs, <0.66 mmol/L and <0.8 mmol/L. The quality of the final model was also analyzed and compared with the quality of the initial model.

Optimization was considered to be achieved for the modified MDQ if it consisted of a smaller number of questions than MDQ-62, if its sensitivity was equal or higher than that of MDQ-62, and if the difference in specificity between MDQ-62 and the modified MDQ was equal to or lower than 20%. Alternatively, optimization was considered to be achieved if the modified MDQ consisted of 15 questions, and if the difference in specificity between MDQ-62 and the modified MDQ was equal to or lower than 10%.

The training sample consisted of the cohort of women with hormone-related conditions, and the test sample consisted of the pregnant women cohort.

The sensitivity and specificity of the modified MDQs were analyzed using the same approach as for MDQ-62.

Correlation coefficients (r) were calculated based on changes from baseline to week 4 in terms of MDQ-62/modified MDQ scores and serum magnesium concentration and analyzed as continuous or dichotomous variables (hypomagnesemia: yes/no). Sensitivity and specificity were estimated using standard formulae [14]. The absolute magnesium concentration was defined as reference.

The study group characteristics (such as demographics) were analyzed using descriptive statistics, and differences were analyzed using the chi square test, unpaired t-test, and non-parametric tests. Statistical significance threshold was set at *p* < 0.05.

## 3. Results

### 3.1. Study Population

A flow chart of the study population is presented in Figure 1 (duplicated records, subjects without serum magnesium test, and subjects <18 years old were excluded from the analysis). In total, 983 participants in the “pregnant women” cohort and 9444 participants in the “women with hormone-related conditions” cohort were eligible for analysis. Participants in the “pregnant women” cohort had a median age of 28.0 years (range 18–52 years) and a mean (SD) serum magnesium level of 0.714 (0.125) mmol/L (range 0.12–1.92 mmol/L). Participants in the “women with hormone-related conditions” cohort had a median age of 44.0 years (range 18–60 years) and a mean (SD) serum magnesium level of 0.776 (0.198) mmol/L (range 0.08–4.08 mmol/L).

**Figure 2:**
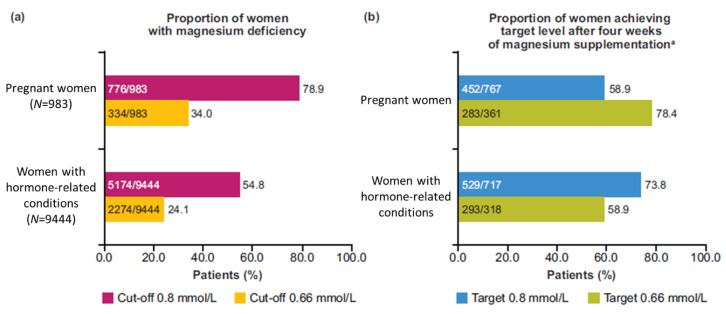
Prevalence and clinical management of magnesium deficiency: (**a**) Proportion of women with magnesium deficiency at Visit 1; (**b**) Proportion of women achieving the target level after four weeks of magnesium supplementation. ^a^ Includes women who had a magnesium serum level below the corresponding target at baseline.

The prevalence of magnesium deficiency assessed by serum levels in pregnant women was 34.0%/78.9% when using 0.66/0.8 mmol/L, respectively, as the cut-off. In women with hormone-related conditions, the prevalence was 24.1%/54.8% when using 0.66/0.8 mmol/L, respectively, as the cut-off (Figure 1). After taking magnesium supplements for four weeks, a large proportion of women in both cohorts was able to achieve a magnesium level above the target cut-offs (Figure 2).

### 3.2. Diagnostic Performance of MDQ-62

The diagnostic performance of MDQ was examined separately in pregnant women and in women with hormone-related conditions using two different cut-offs of magnesium serum levels: 0.8 mmol/L and 0.66 mmol/L. The analysis included 765 pregnant women and 8836 women with hormone-related conditions who both had the results of total serum magnesium test and had filled the MDQ-62 at Visit 1.

In pregnant women, using the cut-off of 0.8 mmol/L, AUROC for MDQ-62 was 0.6301 (standard error [SE] = 0.0251; 95% confidence interval [CI]: 0.5810–0.6792; *p* < 0.0001); using the cut-off of 0.66 mmol/L, AUROC for MDQ-62 was 0.6446 (SE = 0.0210; 95% CI: 0.6036–0.6857; *p* < 0.0001). The positive predictive value of MDQ-62 was slightly better with the cut-off 0.66 mmol/L than that obtained with the cut-off of 0.8 mmol/L (Supplementary Tables S1 and S2).

**Figure 3:**
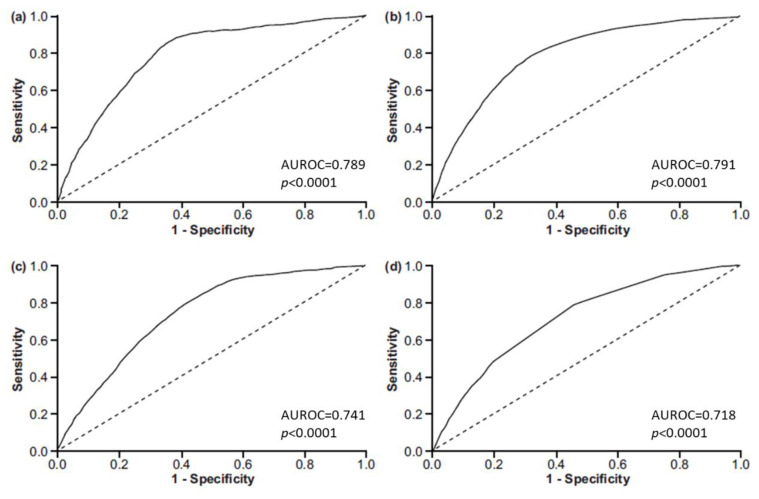
AUROC curves showing the questionnaires′ ability to predict hypomagnesemia in women with hormone-related conditions for: (**a**) MDQ-62 using the cut-off <0.8 mmol/L; (**b**) MDQ-62 using the cut-off <0.66 mmol/L; (**c**) MDQ-23using the cut-off <0.8 mmol/L; (**d**) MDQ-10 using the cut-off <0.66 mmol/L. AUROC, area under the receiver operating characteristic; MDQ, magnesium deficiency questionnaire; The presented *p* values are related to ROC curves implying the H0 hypothesis: AUC = 0.5.

In women with hormone-related conditions, using the cut-off of 0.8 mmol/L, AUROC for MDQ-62 was 0.7893 (SE = 0.0049; 95% CI: 0.7797–0.7990; *p* < 0.0001); using the cut-off of 0.66 mmol/L, AUROC for MDQ-62 was 0.7412 (SE = 0.0057; 95% CI: 0.7300–0.7524; *p* < 0.0001) (Figure 3). For this group, the positive predictive value was slightly worse with the cut-off of 0.66 mmol/L compared to that with the cut-off of 0.8 mmol/L (Supplementary Tables S1 and S2).

In all analyses, an MDQ-62 cut-off ≥51 points provided better specificity, worse sensitivity, better positive likelihood ratio, and worse negative likelihood ratio compared with an MDQ-62 cut-off ≥30. Formally, based on the previously published rough classification system, MDQ-62 had a “poor” (AUROC 0.6–0.7) diagnostic value in pregnant women and a “fair” (AUROC 0.7–0.8) diagnostic value in women with hormone-related conditions [15].

### 3.3. Development of Modified MDQs

Each of the 62 MDQ questions was tested for contribution to the total MDQ score as well as for association with the serum magnesium level in women with hormone-related conditions (training sample), using both cut-offs of <0.8 mmol/L and <0.66 mmol/L. For each of the cut-offs, a modified MDQ was developed that contained questions that showed statistically significant correlation with hypomagnesemia defined as the corresponding cut-off (Supplementary Table S3).

MDQ-23 contained 23 questions that showed a significant correlation with hypomagnesemia defined as <0.8 mmol/L (Supplementary Table S3); its total score range was 0–41, and the optimal cut-off value was >9. MDQ-10 contained 10 questions that showed a significant correlation with hypomagnesemia defined as <0.66 mmol/L (Supplementary Table S4); its total score range was 0–31, and the optimal cut-off value was >5.

The diagnostic performance of the modified MDQs to detect hypomagnesemia at the corresponding cut-offs was tested on the data from pregnant women with symptoms of magnesium deficiency (testing sample).

### 3.4. Diagnostic Performance of MDQ-23 and MDQ-10

The diagnostic performance of MDQ-23 was compared with that of MDQ-62 using the total serum magnesium level cut-off of 0.8 mmol/L (Table 1; Figure 3). MDQ-23 had similar AUROC, higher sensitivity, and lower specificity than MDQ-62 at cut-off ≥30; the 95% CIs overlapped, suggesting that the slight differences were not statistically significant. 

The diagnostic performance of MDQ-10 was compared with that of MDQ-62 using the total serum magnesium level cut-off of 0.66 mmol/L (Table 2; Figure 3). MDQ-10 had a similar AUROC to that of MDQ-62 at score cut-off ≥30 and a higher sensitivity than MDQ-62 in the training sample, but a slightly lower sensitivity in the testing sample and a lower specificity in both samples; the 95% CIs overlapped, suggesting that the detected differences were not statistically significant.

The ability of MDQ-23 and MDQ-10 to detect changes in total serum magnesium level was determined by estimating the correlation between serum magnesium level change and questionnaire score change from Visit 1 to Visit 2. In total, 765 pregnant women and 933 women with hormone-related conditions were included in the analysis (Table 3). In both datasets, statistically significant negative correlations were observed (*p* < 0.0001); however, the correlation coefficients were low (ranging from −0.1667 to −0.2716). For all tested questionnaires, the correlation coefficients were higher for women with hormone-related conditions than for pregnant women.

## 4. Discussion

This study is one of the largest and the most comprehensive real-world studies of magnesium deficiency in women. The study population consisted of pregnant women and women with hormone-related conditions from multiple cities and regions of the Russian Federation, providing a wide geographical coverage and a large sample size (a total of 10,427 women).

Among various magnesium tests, total serum concentration is considered to be the most practical for use in the clinic; however, the prevalence of magnesium deficiency may be underestimated if only blood levels are used [1,2,3,8]. Because of these considerations and the lack of clinical symptoms that specifically indicate magnesium deficiency, serum magnesium tests are rarely ordered and often not reimbursed by insurance companies [3]. There is an unmet need for a non-invasive assessment method that could be used as a surrogate for a clinical diagnosis of magnesium deficiency and at the very least serve as the basis for more detailed laboratory investigations.

This study measured the predictive ability of an existing non-invasive magnesium deficiency screening tool (MDQ-62). It was classified as a “fairly predictive” diagnostic tool for women with hormone-related conditions based on the rough classification of AUROC values [16]; nevertheless, it provided a clinically useful estimate of the magnesium status that may help physicians to identify a possible magnesium deficiency and provide a basis for laboratory testing. This questionnaire may also be used by the general populations to raise or dispel a possible suspicion of magnesium deficiency. The study used the existing MDQ-62 [9] to develop two new short questionnaires (MDQ-23 at cut-off <0.8 mmol/L and MDQ-10 at cut-off <0.66 mmol/L) that have nearly the same diagnostic performance as the original MDQ-62 when assessing likely magnesium deficiency but contain fewer questions and are therefore less time-consuming and easier to administer in routine clinical practice (the initial MDQ-62 and the modified MDQ-23 and MDQ-10 questionnaires with individual questions’ scores are presented below (Table 4).

Having a quick non-invasive cost-free method that could provide further evidence in cases of suspected magnesium deficiency would be very valuable for healthcare providers, especially where there are very few symptoms, and the need for serum testing is otherwise unclear. In this study, the initial long and cumbersome questionnaire was shortened by almost three-fold without losing sensitivity and with a minimal loss of specificity (less than 20%). Both MDQ-23 and MDQ-10 scores showed a statistically significant negative correlation with changes in serum magnesium levels, demonstrating their potential clinical utility in everyday practice as a tool to identify suspected cases of magnesium deficiency.

The symptoms potentially related to magnesium deficiency and included into the MDQ-62 questionnaire are non-specific and may be related to a number of various medical disorders, for instance, B-vitamins deficiencies, low blood calcium, alcohol abuse [16]. Moreover, some of the medical conditions mentioned in the initial MDQ-62 questionnaire (for instance, diabetes) may contribute to some of these symptoms independently of the Mg status [9,17]. Knowing that we did not expect from the beginning a high specificity from the questionnaire based on subjective self-assessment and while establishing research questions, we consciously concentrated our efforts on keeping maximal sensitivity, setting it as a priority in questionnaire optimization. We chose this particular approach to develop a fast non-invasive screening tool to catch most true positive cases implying further laboratory diagnosis verification. The price in this case is usually false positive cases with symptoms/complaints related to other medical conditions and, therefore, a lower specificity of the questionnaire.

MDQ-23 and MDQ-10 showed a slightly worse performance in pregnant women than in women with hormone-related conditions, possibly because some symptoms or signs assessed in the questionnaire may be caused by pregnancy itself rather than by an underlying magnesium deficiency, for example, emotional stress, irritability, or frequent constipation. Another potential reason is that this study used the same questionnaire and cut-offs for pregnant women and women with hormone-related conditions; however, pregnancy may require a different questionnaire design and validation. Finally, pregnancy-related magnesium deficiency may be associated with specific enhanced needs of mother and child, and the natural course of hypomagnesemia may differ in this group of patients. Future studies in pregnant women would be useful to determine whether the questionnaire could be modified further to increase its sensitivity and specificity for this specific population.

The four observational studies (MAGIC, MAGIC2, MAGYN, and MAGYN2) and the analysis presented here were not designed to validate MDQ-62; therefore, it may not be possible to directly extrapolate the results obtained here to the general population without additional validation procedures. One should also bear in mind that the serum levels of magnesium may be misleading, as they do not always accurately reflect the levels in soft tissues and bones; on the other hand, magnesium deficiency can be asymptomatic. Consequently, MDQ and laboratory testing cannot fully replace each other in the assessment of the magnesium status, and the complementary use of both should be considered. It may be advisable to use them step by step, for example to use the questionnaire first to corroborate a suspected deficiency and then follow up with a serum magnesium test.

To our knowledge, MDQ-62 and the two shortened versions presented here are currently the only tools that can be used to assess magnesium deficiency in a non-invasive manner and at no cost. The main advantage of MDQ-23 and MDQ-10 is their relatively high sensitivity (0.8–0.9) in predicting magnesium serum levels <0.8 mmol/L (MDQ-23 score >9) or <0.66 mmol/L (MDQ-10 score >5). Using the modified questionnaires allows clinicians to identify those at a high risk of magnesium deficiency and to verify it further with blood test. The relatively low specificity and therefore potentially high false positive rate are not a major limitation for screening tests, as their main goal is not to miss true positive cases. We believe that MDQs could be very useful in the clinical practice as magnesium deficiency screening tools.

## 5. Conclusions

This analysis determined the sensitivity and specificity of MDQ-62 in identifying patients with suspected magnesium deficiency. Furthermore, we developed two shortened questionnaires (MDQ-23 and MDQ-10), which were non-inferior to MDQ-62. All versions of MDQ showed a better performance in women with hormone-related conditions than in pregnant women.

## Figures and Tables

**Table 1:** Diagnostic performance of MDQ-23 compared with that of MDQ-62 using a serum magnesium level cut-off of 0.8 mmol/L.

	MDQ-62 Score ≥ 30		MDQ-62 Score ≥ 51		MDQ-23 Score> 9	
	Value	95% CI	Value	95% CI	Value	95% CI
**Training Sample: Women with Hormone-Related Conditions**						
**Sensitivity**	0.884	0.874–0.893	0.248	0.236–0.260	0.888	0.879–0.897
**Specificity**	0.616	0.601–0.631	0.938	0.930–0.945	0.524	0.508–0.539
**AUROC**	0.789	0.780–0.799	0.789	0.780–0.799	0.791	0.781–0.800
***p* value**	<0.001				<0.001	
**Testing Sample: Pregnant Women with Symptoms of Magnesium Deficiency**						
**Sensitivity**	0.845	0.815–0.872	0.283	0.248–0.320	0.864	0.835–0.890
**Specificity**	0.235	0.165–0.316	0.864	0.793–0.917	0.197	0.133–0.275
**AUROC**	0.630	0.581–0.679	0.630	0.581–0.679	0.610	0.560–0.659

CI, confidence interval; the presented *p* values are related to ROC curves implying the H0 hypothesis: AUC = 0.5.

**Table 2:** Diagnostic performance of MDQ-10 compared with that of MDQ-62 using a serum magnesium level cut-off of 0.66 mmol/L.

	MDQ-62 Score ≥ 30		MDQ-62 Score ≥ 51		MDQ-10 Score> 5	
	Value	95% CI	Value	95% CI	Value	95% CI
**Training Sample: Women with Hormone-Related Conditions**						
**Sensitivity**	0.927	0.915–0.938	0.310	0.290–0.331	0.940	0.929–0.950
**Specificity**	0.427	0.415–0.439	0.881	0.873–0.889	0.260	0.250–0.271
**AUROC**	0.741	0.730–0.752	0.741	0.730–0.752	0.718	0.706–0.730
***p* value**	<0.001				<0.001	
**Testing Sample: Pregnant Women with Symptoms of Magnesium Deficiency**						
**Sensitivity**	0.887	0.843–0.922	0.358	0.301–0.418	0.869	0.823–0.906
**Specificity**	0.200	0.165–0.238	0.798	0.760–0.833	0.171	0.139–0.207
**AUROC**	0.645	0.604–0.686	0.645	0.604–0.686	0.610	0.568–0.652

The presented *p* values are related to ROC curves implying the H0 hypothesis: AUC = 0.5.

**Table 3:** Correlation between changes from Visit 1 to Visit 2 for total serum magnesium level and for initial (MDQ-62) and modified (MDQ-23 and MDQ-10) questionnaire scores.

	Women with Hormone-Related Conditions (*N* = 933)		Pregnant Women with Symptoms of Magnesium Deficiency (*N* = 765)	
	*r*	*p Value*	*r*	*p Value*
MDQ-62	−0.2716	<0.0001	−0.2253	<0.0001
MDQ-23	−0.2890	<0.0001	−0.1729	<0.0001
MDQ-10	−0.2072	<0.0001	−0.1667	<0.0001

r, Pearson correlation coefficient. The presented *p* values are related to the Pearson correlation coefficient implying the H0 hypothesis: r = 0.

**Table 4:** Initial (MDQ-62) and modified (MDQ-23, MDQ-10) questionnaires with individual questions′ scores.

Question Number in MDQ-62	Question	Question Score in MDQ-62	Question Score in MDQ-23	Question Score in MDQ-10
Q1	Excessive emotional stress	2	2	3
Q2	Irritable, or easily provoked to anger	3	2	3
Q3	Restless, or hyperactive	2	-	-
Q4	Easily startled by sound or light	4	-	-
Q5	Insomnia	2	1	3
Q6	Chronic headache or migraine	3	-	-
Q7	Convulsions	2	2	-
Q8	Tremor or shakiness in the hands	3	-	-
Q9	Fine, barely noticeable muscle twitching around your eyes, facial muscles, or other muscles of your body	3	1	-
Q10	Muscle spasms	3	-	-
Q11	Muscle spasms in hands or feet	3	2	-
Q12	Gag or choke from spasms in your esophagus (food tube)	4	2	-
Q13	Asthma, short breathing, rales	3	-	-
Q14	Emphysema, chronic bronchitis, or high respiratory rate (tachypnea)	2	-	-
Q15	Osteoporosis	5	-	-
Q16	Kidney stone disease (urolithiasis)	3	-	3
Q17	Chronic kidney disease	2	2	-
Q18	Diabetes	4	-	-
Q19	Hyperfunction of the thyroid or parathyroid gland	3	-	-
Q20	High blood pressure	3	-	-
Q21	Mitral valve prolapse (“floppy heart valve”)	4	4	4
Q22	Tachycardia, irregular heartbeat, or arrhythmia	3	-	-
Q23	Chronic bowel disease, ulcerative colitis, Crohn′s disease or irritable bowel syndrome	3	-	-
Q24	Frequent diarrhea or constipation	3	2	3
Q25	Suffer from premenstrual syndrome or menstrual cramps	3	-	-
Q26	Pregnant or recently pregnant	2	-	-
Q27	Take Digitalis (Digoxin)	3	-	-
Q28	Take any kind of diuretic	5	1	-
Q29	Recent radiation therapy or other type of radiation exposure	5	-	-
Q30	Have more than seven alcohol drinks weekly	4	1	-
Q31	Problems with excessive alcohol intake	3	-	-
Q32	Take more than three portions of caffeine-containing drinks daily	2	-	-
Q33	Consumption of sugar-containing products	2	-	-
Q34	Crave carbohydrates and/or chocolate	2	2	-
Q35	Crave salt and/or salt products	2	-	-
Q36	Eat a high-processed food/fast food diet	2	-	-
Q37	Eat a diet low in greens, leafy vegetables, seeds, and fresh fruit	2	1	-
Q38	Eat a low-protein diet	2	-	-
Q39	Presence of undigested food or fat in feces	2	-	-
Q40	High blood pressure or pre-eclampsia in previous pregnancy	4	-	-
Q41	Chronic fatigue	2	1	-
Q42	Muscle weakness	2	2	-
Q43	Feeling of cold hands and/or feet	2	-	-
Q44	Numbness in face, hands, or feet	2	-	-
Q45	Persistent tingling in the body	2	2	3
Q46	Feeling of chronic indifference or apathy	2	-	4
Q47	Poor memory	2	-	-
Q48	Loss of concentration	2	1	-
Q49	Anxiety	3	2	-
Q50	Chronic depression for no apparent reason	2	2	-
Q51	Feelings of disorientation as to time or place	2	-	-
Q52	Feeling depressed, lack of personal identity	2	-	-
Q53	Hallucinations	2	-	-
Q54	Feeling of persecution and hostility from others	2	-	-
Q55	Pale and puffy face or poor, bad complexion	2	-	-
Q56	Loss of considerable sexual energy or vitality	2	2	-
Q57	Been told by your attending doctor that your blood calcium is low	2	-	3
Q58	Been told by your attending doctor that your blood potassium is low	3	-	-
Q59	Take calcium supplements regularly without magnesium	2	3	2
Q60	Take iron or zinc supplements regularly without magnesium	2	-	-
Q61	Chronic fluoride intake	2	-	-
Q62	Frequently use antibiotics, steroids, oral contraceptives, indomethacin, cisplatin, amphotericin B, cholestyramine, synthetic estrogens	3	1	-

## Supplementary Materials

The following are available online at https://www.mdpi.com/2072-6643/12/7/2062/s1, Table S1: Diagnostic utility of MDQ using a serum magnesium level cut-off of 0.8 mmol/L, Table S2: Diagnostic utility of MDQ using a serum magnesium level cut-off of 0.66 mmol/L, Table S3: Development of MDQ-23, Table S4: Development of MDQ-10.
