# Supplementary material for: Magnesium Deficiency Questionnaire: A New Non-Invasive Magnesium Deficiency Screening Tool Developed Using Real-World Data from Four Observational Studies
Source: Nutrients. 2020 Jul 11;12(7):2062. doi: 10.3390/nu12072062 (PMC7400907; doi:10.3390/nu12072062)
Supplement: Supplementary file 1 [file nutrients-12-02062-s001.pdf]

Supplementary Table S1. Diagnostic utility of MDQ using serum magnesium level cut-off of 0.8 mmol/L.

	Pregnant women (N=765)				Women with hormone-related conditions (N=8836)			
	MDQ-62 score ≥30		MDQ≥51		MDQ≥30		MDQ≥51	
	Value	95% CI	Value	95% CI	Value	95% CI	Value	95% CI
**Sensitivity**	0.845	0.815–0.872	0.283	0.248–0.320	0.884	0.874–0.893	0.248	0.236–0.260
**Specificity**	0.235	0.165–0.316	0.864	0.793–0.917	0.616	0.601–0.631	0.938	0.930–0.945
**Positive predictive value**[a]	0.841	0.774–0.869	0.909	0.858–0.922	0.729	0.716–0.746	0.824	0.805–0.833
**Negative predictive value**[a]	0.240	0.203–0.323	0.201	0.174–0.305	0.820	0.806–0.829	0.517	0.500–0.549
**Positive likelihood ratio**	1.105	0.999–1.221	2.074	1.326–3.242	2.300	2.209–2.394	4.010	3.523–4.564
**Negative likelihood ratio**	0.659	0.461–0.943	0.830	0.764–0.903	0.189	0.174–0.205	0.802	0.787–0.816
**AUROC**	0.630[b]	0.581–0.679	0.630[b]	0.581–0.679	0.789[b]	0.780–0.799	0.789[b]	0.780–0.799

[a]Note that positive and negative predictive values are influenced by the prevalence of condition in the population that is being tested. [b]p-values for AUROC were $p<0.0001$.

CI, confidence interval; MDQ, magnesium deficiency questionnaire.

Supplementary Table S2. Diagnostic utility of MDQ using serum magnesium level cut-off of 0.66 mmol/L.

	Pregnant women (N=765)				Women with hormone-related conditions (N=8836)			
	MDQ≥30		MDQ≥51		MDQ≥30		MDQ≥51	
	Value	95% CI	Value	95% CI	Value	95% CI	Value	95% CI
**Sensitivity**	0.887	0.843–0.922	0.358	0.301–0.418	0.927	0.915–0.938	0.310	0.290–0.331
**Specificity**	0.200	0.165–0.238	0.798	0.760–0.833	0.427	0.415–0.439	0.881	0.873–0.889
**Positive predictive value**[a]	0.382	0.329–0.482	0.497	0.442–0.560	0.321	0.311–0.360	0.433	0.415–0.457
**Negative predictive value**[a]	0.760	0.685–0.798	0.690	0.633–0.737	0.952	0.944–0.954	0.814	0.799–0.825
**Positive likelihood ratio**	1.108	1.042–1.178	1.774	1.400–2.248	1.617	1.579–1.656	2.615	2.385–2.866
**Negative likelihood ratio**	0.567	0.389–0.825	0.805	0.729–0.888	0.171	0.146–0.201	0.783	0.759–0.807
**AUROC**	0.645[b]	0.604–0.686	0.645[b]	0.604–0.686	0.741[b]	0.730–0.752	0.741[b]	0.730–0.752

[a]Note that positive and negative predictive values are influenced by the prevalence of condition in the population that is being tested. [b]p-values for AUROC were $p<0.0001$.

CI, confidence interval; MDQ, magnesium deficiency questionnaire.

Supplementary Table S3. Development of MDQ-23.

Questions included in the original MDQ-62 questionnaire were tested for contribution to the overall score in a regression model based on magnesium below 0.8 mmol/L (Yes/no). In MDQ-62, each question contributed 2–5 points to the overall score; a score of ≥51 points indicated a 'highly probable' magnesium deficiency. In MDQ-23, each selected question was assigned a new value of 1–4 points that contributed to the overall score; a score of >9 points indicated magnesium deficiency.

Question number in MDQ-62	Question	Question score in MDQ-62	**Regression coefficient**	**Standard error**	**p-value**	Question score in MDQ-23
Q1	Excessive emotional stress	2	0.424	0.055	<0.0001	2
Q2	Irritable, or easily provoked to anger	3	0.492	0.062	<0.0001	2
Q3	Restless, or hyperactive	2				-
Q4	Easily startled by sound or light	4				-
Q5	Insomnia	2	0.302	0.051	<0.0001	1
Q6	Chronic headache or migraine	3				-
Q7	Convulsions	2	0.324	0.065	<0.0001	2
Q8	Tremor or shakiness in the hands	3				-
Q9	Fine, barely noticeable muscle twitching around your eyes, facial muscles, or other muscles of your body	3	0.311	0.059	<0.0001	1
Q10	Muscle spasms	3				-

Q11	Muscle spasms in hands or feet	3	0.332	0.059	<0.0001	2
Q12	Gag or choke from spasms in your oesophagus (food tube)	4	0.503	0.088	<0.0001	2
Q13	Asthma, shortbreathing, rales	3				-
Q14	Emphysema, chronic bronchitis, or high respiratory rate (tachypnea)	2				-
Q15	Osteoporosis	5				-
Q16	Kidney stone disease (urolithiasis)	3				-
Q17	Chronic kidney disease	2	0.452	0.068	<0.0001	2
Q18	Diabetes	4				-
Q19	Hyperfunction of the thyroid or parathyroid gland	3				-
Q20	High blood pressure	3				-
Q21	Mitral valve prolapse (‘floppy heart valve’)	4	0.824	0.139	<0.0001	4
Q22	Tachycardia, irregular heartbeat or arrhythmia	3				-
Q23	Chronic bowel disease, ulcerative colitis, Crohn’s disease or irritable bowel syndrome	3				-
Q24	Frequent diarrhea or constipation	3	0.513	0.079	<0.0001	2
Q25	Suffer from premenstrual syndrome or menstrual cramps	3				-
Q26	Pregnant or recently pregnant	2				-
Q27	Take Digitalis (Digoxin)	3				-
Q28	Take any kind of diuretic	5	0.284	0.052	<0.0001	1

Q29	Recent radiation therapy or other type of radiation exposure	5				-
Q30	Have more than 7 alcohol drinks weekly	4	0.277	0.051	<0.0001	1
Q31	Problems with excessive alcohol intake	3				-
Q32	Take more than 3 portions of caffeine-containing drinks daily	2				-
Q33	Consumption of sugar-containing products	2				-
Q34	Crave carbohydrates and/or chocolate	2	0.352	0.056	<0.0001	2
Q35	Crave salt and/or salt products	2				-
Q36	Eat a high processed food/fast food diet	2				-
Q37	Eat a diet low in green, leafy vegetables, seeds and fresh fruit	2	0.257	0.056	<0.0001	1
Q38	Eat a low protein diet	2				-
Q39	Presence of undigested food or fat in feces	2				-
Q40	High blood pressure or pre-eclampsia in previous pregnancy	4				-
Q41	Chronic fatigue	2	0.275	0.055	<0.0001	1
Q42	Muscle weakness	2	0.473	0.053	<0.0001	2
Q43	Feeling of cold hands and/or feet	2				-
Q44	Numbness in face, hands or feet	2				-
Q45	Persistent tingling in body	2	0.487	0.086	<0.0001	2

Q46	Feeling of chronic indifference or apathy	2				-
Q47	Poor memory	2				-
Q48	Loss of concentration	2	0.237	0.053	<0.0001	1
Q49	Anxiety	3	0.383	0.051	<0.0001	2
Q50	Chronic depression for no apparent reason	2	0.528	0.066	<0.0001	2
Q51	Feelings of disorientation as to time or place	2				-
Q52	Feeling depressed, lack of personal identity	2				-
Q53	Hallucinations	2				-
Q54	Feeling of persecution and hostility from others	2				-
Q55	Pale and puffy face or poor, bad complexion	2				-
Q56	Loss of considerable sexual energy or vitality	2	0.368	0.052	<0.0001	2
Q57	Been told by your attending doctor that your blood calcium is low	2				-
Q58	Been told by your attending doctor that your blood potassium is low	3				-
Q59	Take calcium supplements regularly without magnesium	2	0.537	0.078	<0.0001	3
Q60	Take iron or zinc supplements regularly without magnesium	2				-
Q61	Chronic fluoride intake	2				-

Q62	Frequently use antibiotics, steroids, oral contraceptives, indomethacin, cisplatin, amphotericin B, cholestyramine, synthetic oestrogens	3	0.313	0.061	<0.0001	1

MDQ, magnesium deficiency questionnaire.

Supplementary Table S4. Development of MDQ-10.

Questions included in the original MDQ-62 questionnaire were tested for contribution to the overall score in a regression model based on magnesium below 0.66 mmol/L (Yes/no). In MDQ-62, each question contributed 2–5 points to the overall score; a score of ≥51 points indicated a ‘highly probable’ magnesium deficiency. In MDQ-10, each selected question was assigned a new value of 1–4 points that contributed to the overall score; a score of >5 points indicated magnesium deficiency.

Question number in MDQ-62	Question	Question score in MDQ-62	**Regression coefficient**	**Standard error**	**p-value**	Question score in MDQ-10
Q1	Excessive emotional stress	2	0.554	0.065	<0.0001	3
Q2	Irritable, or easily provoked to anger	3	0.684	0.079	<0.0001	3
Q3	Restless, or hyperactive	2				-
Q4	Easily startled by sounds or lights	4				-
Q5	Insomnia	2	0.552	0.058	<0.0001	3
Q6	Chronic headaches or migraines	3				-
Q7	Convulsions	2				-
Q8	Tremor or shakiness in the hands	3				-
Q9	Fine, barely noticeable muscle twitching around your eyes, facial muscles, or other muscles of your body	3				-
Q10	Muscle spasms	3				-
Q11	Muscle spasms in hands or feet	3				-

Q12	Gag or choke from spasms in your oesophagus (food tube)	4				-
Q13	Asthma, shortbreathing, rales	3				-
Q14	Emphysema, chronic bronchitis, or high respiratory rate (tachypnea)	2				-
Q15	Osteoporosis	5				-
Q16	Kidney stone disease (urolithiasis)	3	0.543	0.103	<0.0001	3
Q17	Chronic kidney disease	2				-
Q18	Diabetes	4				-
Q19	Hyperfunction of the thyroid or parathyroid gland	3				-
Q20	High blood pressure	3				-
Q21	Mitral valve prolapse (‘floppy heart valve’)	4	0.793	0.121	<0.0001	4
Q22	Tachycardia, irregular heartbeat or arrhythmia	3				-
Q23	Chronic bowel disease, ulcerative colitis, Crohn’s disease or irritable bowel syndrome	3				-
Q24	Frequent diarrhea or constipation	3	0.554	0.073	<0.0001	3
Q25	Suffer from premenstrual syndrome or menstrual cramps	3				-
Q26	Pregnant or recently pregnant	2				-
Q27	Take Digitalis (Digoxin)	3				-
Q28	Take any kind of diuretic	5				-

Q29	Recent radiation therapy or other type of radiation exposure	5				-
Q30	Have more than 7 alcohol drinks weekly	4				-
Q31	Problems with excessive alcohol intake	3				-
Q32	Take more than 3 portions of caffeine-containing drinks daily	2				-
Q33	Consumption of sugar-containing products	2				-
Q34	Crave carbohydrates and/or chocolate	2				-
Q35	Crave salt and/or salt products	2				-
Q36	Eat a high processed food/fast food diet	2				-
Q37	Eat a diet low in green, leafy vegetables, seeds and fresh fruit	2				-
Q38	Eat a low protein diet	2				-
Q39	Presence of undigested food or fat in feces	2				-
Q40	High blood pressure or pre-eclampsia in previous pregnancy	4				-
Q41	Chronic fatigue	2				-
Q42	Muscle weakness	2				-
Q43	Feeling of cold hands and/or feet	2				-
Q44	Numbness in face, hands or feet	2				-
Q45	Persistent tingling in body	2	0.571	0.076	<0.0001	3

Q46	Feeling of chronic indifference or apathy	2	0.766	0.069	<0.0001	4
Q47	Poor memory	2				-
Q48	Loss of concentration	2				-
Q49	Anxiety	3				-
Q50	Chronic depression for no apparent reason	2				-
Q51	Feelings of disorientation as to time or place	2				-
Q52	Feeling depressed, lack of personal identity	2				-
Q53	Hallucinations	2				-
Q54	Feeling of persecution and hostility from others	2				-
Q55	Pale and puffy face or poor, bad complexion	2				-
Q56	Loss of considerable sexual energy or vitality	2				-
Q57	Been told by your attending doctor that your blood calcium is low	2	0.571	0.076	<0.0001	3
Q58	Been told by your attending doctor that your blood potassium is low	3				-
Q59	Take calcium supplements regularly without magnesium	2	0.766	0.069	<0.0001	2
Q60	Take iron or zinc supplements regularly without magnesium	2				-
Q61	Chronic fluoride intake	2				-

Q62	Frequently use antibiotics, steroids, oral contraceptives, indomethacin, cisplatin, amphotericin B, cholestyramine, synthetic oestrogens	3				-

MDQ, magnesium deficiency questionnaire.
